# HPV and lung cancer: A systematic review and meta‐analysis

**DOI:** 10.1002/cnr2.1350

**Published:** 2021-02-23

**Authors:** Julia Karnosky, Wolfgang Dietmaier, Helge Knuettel, Viola Freigang, Myriam Koch, Franziska Koll, Florian Zeman, Christian Schulz

**Affiliations:** ^1^ Klinik und Poliklinik für Innere Medizin II, Bereich Pneumologie Klinikum der Universität Regensburg Regensburg Germany; ^2^ Institut für Pathologie Universität Regensburg Regensburg Germany; ^3^ Universitätsbibliothek Universität Regensburg Regensburg Germany; ^4^ Klinik und Poliklinik für Unfallchirurgie Klinikum der Universität Regensburg Regensburg Germany; ^5^ Zentrum für Klinische Studien Klinikum der Universität Regensburg Regensburg Germany

**Keywords:** carcinogenesis, HPV, lung cancer, meta‐analysis

## Abstract

**Background:**

Lung cancer has emerged as a global public health problem and is the most common cause of cancer deaths by absolute cases globally. Besides tobacco, smoke infectious diseases such as human papillomavirus (HPV) might be involved in the pathogenesis of lung cancer. However, data are inconsistent due to differences in study design and HPV detection methods.

**Aim:**

A systematic meta‐analysis was performed to examine the presence of HPV‐infection with lung cancer.

**Methods and Results:**

All studies in all languages were considered for the search concepts “lung cancer” and “HPV” if data specific to HPV prevalence in lung cancer tissue were given. This included Journal articles as well as abstracts and conference reports. As detection method, only HPV PCR results from fresh frozen and paraffin‐embedded tissue were included. Five bibliographic databases and three registers of clinical trials including MEDLINE, Embase, Cochrane Library, and ClinicalTrials.gov were searched through February 2020. A total 4298 publications were identified, and 78 publications were selected, resulting in 9385 included lung cancer patients. A meta‐analysis of 15 case‐control studies with n = 2504 patients showed a weighted overall prevalence difference of 22% (95% CI: 12%‐33%; *P* < .001) and a weighted overall 4.7‐fold (95% CI: 2.7‐8.4; *P* < .001) increase of HPV prevalence in lung cancer patients compared to controls. Overall, HPV prevalence amounted to 13.5% being highest in Asia (16.6%), followed by America (12.8%), and Europe (7.0%). A higher HPV prevalence was found in squamous cell carcinoma (17.9%) compared to adenocarcinoma (*P* < .01) with significant differences in geographic patterns. HPV genotypes 16 and 18 were the most prevalent high‐risk genotypes identified.

**Conclusion:**

In conclusion, our review provides convincing evidence that HPV infection increases the risk of developing lung cancer.

**Abbreviations**
AC: adeno carcinoma
AhR: aryl hydrocarbon receptor
ALK: anaplastic lymphoma kinase
ARI: absolute risk increase
cIAP‐2: baculoviral IAP repeat‐containing protein3
E6: E6 oncoprotein of human papillomavirus
E7: E7 oncoprotein of human papillomavirus
EGFR: epidermal growth factor receptor
Embase: biomedical and pharmacological bibliographic database
EU: European Union
FHIT: fragile histidine triad protein
HER‐2: receptor tyrosine‐protein kinase erbB‐2
HIF‐1α: hypoxia‐inducible factor 1‐alpha
HPV: human papillomavirus
hTERT: human telomerase reverse transcriptase
IL: interleukin
MCL1: induced myeloid leukemia cell differentiation protein
MEDLINE: U.S. National Library of Medicine
NHS: National Health Service
p53: cellular tumor antigen p53
PCR: polymerase chain reaction
PD: prevalence difference
PR: prevalence ratio
pRb: retinoblastoma protein
ROS1: proto‐oncogene tyrosine‐protein kinase ROS
SCC: squamous cell carcinoma
VEGF: vascular endothelial growth factor
WHO: World Health Organization

## 1 INTRODUCTION

Lung cancer is estimated to be the leading cause of cancer‐related mortality worldwide, with 2.1 million new lung cancer cases and 1.8 million predicted deaths worldwide in 2018.^1^ Although smoking by far has been identified as the most important risk factor in lung cancer, other interactions with environmental and/or genetic risk factors as well as infectious diseases have been identified to contribute to the pathogenesis of lung cancer as well.

Viral infections, such as human papillomavirus (HPV) infections have been reported to be an important risk factor of cervical cancer if genotypes with a high oncogenic risk are found. Since the first identification of human papillomavirus, more than 200 different subtypes have been identified They are classified into high‐risk HPV types (16, 18, 31, 33, 39, 45, 51, 52, and 58) and low‐risk HPV types (6, 11, 42, 43, and 44).^2^ In some other publications, a differentiation between high‐, intermediate‐, and low‐risk HPV types can be found.^3^ Although HPV infection has been identified as a potential contributor to the pathogenesis in lung cancer in certain populations, such as never smokers, its role still remains controversial. Numerous tests, such as nucleic acid amplification, HPV DNA‐based in situ hybridization, immunohistochemistry, and cytology are available for HPV‐testing and screening.^4^, ^5^ The current study focused on the prevalence of HPV infections in lung cancer patients in which HPV detection was performed by means of PCR from fresh frozen and/or paraffin‐embedded tissue to first minimize differences in HPV prevalence due to methodological bias and second to rely on the method with the highest sensitivity to detect HPV positivity, which has been proven to have the highest sensitivity in earlier studies.^4^, ^5^ We conducted and report here a systematic review on the issue above.

## 2 METHODS

The methods of the systematic review and meta‐analysis were specified in advance and published in a protocol registered with PROSPERO. Reporting of this meta‐analysis was done according to the recommendation of Stroup et al for reporting observational studies.^6^

### 2.1 Evidence search and meta‐analysis

The digital databases Embase (via Ovid, 1974‐present), MEDLINE (via Ovid, 1946‐present), Cochrane Library (Cochrane Database of Systematic Reviews, Database of Abstracts of Reviews of Effect, Cochrane Central Register of Controlled Trials, Health Technology Assessment Database, NHS Economic Evaluation Database; from inception to present), and Science Citation Index Expanded (Web of Science, 1965‐present), as well as the search engine Google Scholar (using Anne‐Wil Harzing's “Publish or Perish” program available from https://harzing.com/resources/publish-or-perish), were searched. From Google Scholar, only the first 200 records (initial search on April 25, 2018; no date limit) and the first 100 records (update search on February 6, 2020; date limit years 2018‐2020) were downloaded (default sort order). In addition, WHO's International Clinical Trials Registry Platform, ClinicalTrials.gov, and the EU Clinical Trials Register were searched for completed studies. All searches were last updated on February 6, 2020. We deviated from the protocol; in that, we did not search the German Clinical Trials Register due to its search interface giving erroneous results. An initial, sensitive search strategy for the concepts “lung cancer” AND “HPV” was developed for Embase by a medical librarian in cooperation with subject matter experts and then adapted to the other databases. Controlled terms from the databases' thesauri and a broad range of synonyms were used. No limits such as for study type, publication type, publication date, or language were applied. Search strategies that allow for reproducing the searches are documented in Appendix 1. Database searches were carried out by a medical librarian. The reference lists of included studies and of relevant systematic reviews were screened for additional studies. Records from the database searches were imported into Endnote software for deduplication. Screening by title and abstract and subsequent full‐text assessment were done in Covidence. Titles and abstracts of the publications were analyzed by three independent reviewers (F.K., J.K., and C.S.) for relevance and matching inclusion criteria. Analysis of the publications was done according to prespecified inclusion and exclusion criteria.

All studies reporting HPV prevalence in primary lung cancer cases in adults were included. Case reports were excluded. As detection method, only PCR from fresh frozen and/or paraffin‐embedded tissue were included. All types of tissue sampling method were included. HPV detection in archival tumor tissue was included as well. Only studies that provide data specific to HPV prevalence in lung cancer tissue were included. No exclusions were made based on language. Journal articles as well as abstracts and conference reports were included if they met the inclusion criteria. Journal articles that reported about not only cases of HPV detection in primary lung cancer but, for example, in head and neck cancer as well, were included but only the data of the primary lung cancer group were extracted.

### 2.2 Statistical analysis

The total number of cases, as well as the number of positive and negative HPV detections, was collected from the selected records, and HPV prevalences were calculated by means of the extracted patient data. The Chi‐squared‐test of independence was used to analyze whether prevalence rates differ between continents. Furthermore, a meta‐analysis was performed on a small subset of case‐control studies regarding HPV prevalence. Prevalence difference (PD) and prevalence ratio (PR) both accompanied with the corresponding 95% confidence intervals were estimated for each study. To estimate PR in studies with no HPV positive cases, 0.5 was added to each cell of the 2 × 2 table as usually recommended. Random‐effect models were used to determine the weighted averages of PD and PR while allowing for heterogeneity of effects. The Q‐statistic as a measurement for between‐study heterogeneity and I^2^‐statistic for quantification of the proportion of total variation due to heterogeneity were calculated. Analyses were performed using R version 4.0.3 (The R Foundation for Statistical Computing), the meta‐analysis by using the metafor package. For all comparisons, a *P* value <.05 was considered as statistically significant.

## 3 RESULTS

### 3.1 Evidence Search

**FIGURE 1:**
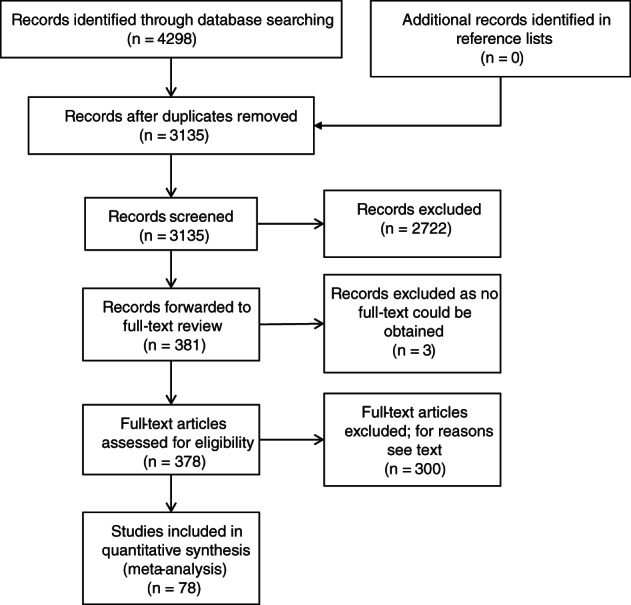
PRISMA flowchart of selected and analyzed studies

The database searches were last updated on February 6, 2020 and yielded a total of 4525 records. Following deduplication, 3135 publications were evaluated on relevance for the research question. A total of 2754 of the titles and abstracts did not relate to the current research and were excluded. In summary, 381 publications were entered into the full text review. Full texts of three possibly relevant publications could not be obtained despite some efforts and therefore were not available^7^, ^8^, ^9^ for further analyses. The remaining 378 full‐texts were assessed for eligibility. After applying the inclusion and exclusion criteria, 78 publications were included in this systematic review. Reasons for exclusion were as follows: No PCR data were reported (n = 80). HPV detection method was not detailed (n = 2). Duplication of the data (n = 22). Case reports (n = 9). Corrections and/or comments on screened publications (n = 15). Systematic reviews and meta‐analysis (n = 29). Overview articles (n = 29). HPV detection was not done in lung biopsies (n = 32). HPV prevalence analyzed in cancers other than lung cancer or on metastasis (n = 6). Missing data on HPV prevalence (n = 40). Same patients in separate publications (n = 7). Same information in different languages (n = 4). Abstract published in a different journal than the full text (n = 12). HPV prevalence in lung cancer in special patient groups, for example, patients after lung transplantation, immunocompromised patients, butchers, and respiratory papillomatosis (n = 7). Unfinished studies (n = 4). No data on sampling method were provided (n = 2). This review process was performed according to the PRISMA statement. Figure 1 depicts the flow of citations reviewed for the meta‐analysis.

A total of 15 publications were case‐control studies, in which normal lung tissue was used as a control (see Table 1).

**TABLE 1:** Included case‐control studies

Author	Year	No. of cases	No. of positive cases	HPV prevalence cases [%]	No. of controls	No. of positive controls	HPV prevalence controls [%]
Carpagnano et al^10^	2011	89	12	13.5	68	0	0.0
Cheng et al^11^	2004	141	54	38.3	60	1	1.7
Cheng et al^12^	2001	141	77	54.6	60	16	26.7
Eberlein‐Gonska et al^13^	1992	55	3	5.5	15	0	0.0
Fan et al^14^	2015	262	22	8.4	19	0	0.0
Galvan et al^15^	2012	85	0	0	100	0	0.0
Gatta et al^16^	2012	50	2	4.0	23	2	8.7
Li et al^17^	1995	50	16	32.0	22	0	0.0
Lu et al^18^	2016	72	33	45.8	54	2	3.7
Nadji et al^19^	2007	129	33	25.6	89	8	9.0
Robinson et al^20^	2016	70	9	12.9	10	1	10.0
Wang et al^21^	2008	313	138	44.1	96	4	4.2
Wang et al^22^	2010	45	19	42.2	16	0	0
Yu et al^23^	2015	180	100	55.6	110	7	6.4
Zhang^24^	2009	68	30	44.1	12	1	8.3
Total		1750	548	31.3	754	42	5.6

The studies were stratified according to the geographical region in which the patients lived. There were 36 studies on patients from Asia, 25 studies on European patients, and 17 studies carried out on the American continent. The countries most represented were Japan (n = 11), China (n = 11), United States (n = 9), and Italy (n = 5). Three studies from Germany met the inclusion criteria. Six studies were done in multiple countries with the information summarized in one publication. Most of the publications were written in English (n = 73). The other publications were published in Chinese (n = 3), French (n = 1), and German (n = 1). In order to get information on as many cases as possible not only journal articles but every type of available study was included. Of the 78 included publications, 67 were journal articles. Of the remaining publications, six were abstracts, three were poster presentations, and two were meeting abstracts.

### 3.2 Patients characteristics

A total of 9385 lung cancer patients were included into this systematic review. Twenty‐eight studies provided data on the patients' age. The average age of all studies ranged from 51.6 to 70 years. Information on patients' gender was available in 52 out of the 78 studies. Those studies included 6326 patients. Of them, 62.8% were male and 37.2% were female, respectively. The percentage of male patients ranged from 0.0% to 91%. Smoking behavior was detailed in 31 of the studies. There were 3577 current or former smokers, 1958 never smokers, and in 3850 cases, no information on smoking status was available. The rate of smokers was 64.6% and ranged from 0% to 100%.

### 3.3 Meta‐analysis of 15 case‐control studies

**FIGURE 2:**
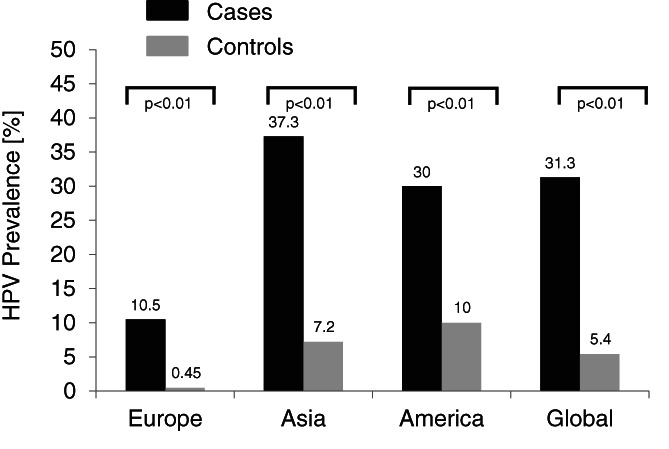
Overall HPV prevalence in case‐control studies as well as divided by different continents. There was a significant difference between the HPV prevalence in cases and controls overall as well as in Europe and Asia (*P* < .01)

**FIGURE 3:**
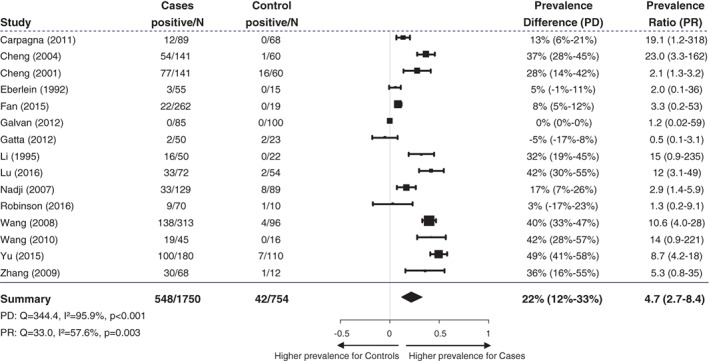
Forest plot demonstrating prevalence difference and prevalence ratio of HPV detection in lung cancer patients compared to control patients without lung cancer. PR of studies with no HPV positive cases in one of the groups was calculated by adding 0.5 to each cell of the 2 × 2 table. Random effect models were used to calculate summary statistics

A total of 1750 lung cancer cases and 754 controls were analyzed, which were derived from 15 case‐control studies (Table 1). One of them is from America, 10 are from Asia, and four from Europe. The overall HPV prevalence was detected to be 31.3% (548/1750) in the lung cancer group and 5.5% (42/754) in the control group (*P* < .001). Figure 2 shows the HPV prevalence derived from case‐control studies as well as divided by different continents. Comparing HPV prevalence of patients with lung cancer and controls in a meta‐analysis, using the 15 case‐control studies with a total of 2504 patients, a higher prevalence could be found for the lung cancer patients for prevalence difference (PD = 0.22; 95%‐CI, 0.12‐0.33; *P* < .001) as well as prevalence ratio (PR = 4.7; 95% CI, 2.7‐8.4; *P* < .001). A forest plot summarizing the data and the effect estimates is shown in Figure 3. Due to the large confidence intervals of the PRs, only PDs are presented graphically. According to the Q‐statistic, a significant difference in between‐study heterogeneity could be identified [PD: Q(*df* = 14) = 344.4, *I* ^2^ = 95.94%, *P* < .001; PR: Q(*df* = 14) = 33.0, *I* ^2^ = 57.6% (PR), *P* = .003].

### 3.4 HPV prevalence

**FIGURE 4:**
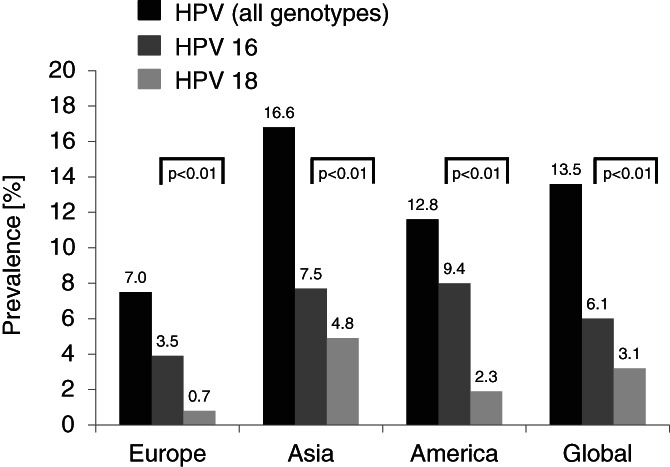
Overall HPV, HPV 16, and HPV 18 prevalence in all analyzed lung cancer cases and between analyzed continents. The highest HPV prevalence was detected in Asia followed by The Americas and Europe. Overall and on all three continents the prevalence of HPV 16 was significantly higher than for HPV 18. The highest HPV 16 prevalence was detected in The Americas followed by Asia and Europe. The highest HPV 18 prevalence was found in Asia followed by The Americas and finally Europe

Of all included patients with lung cancer (n = 9385), HPV was detected to be positive in 1268 cases. The overall HPV prevalence was calculated to be 13.5%. The highest HPV prevalence was detected in Asia with 16.6% (*P* < .01 vs America and Europe), followed by The Americas (12.8%; *P* < .01 vs Europe) and Europe (7.0%). The highest HPV 16 prevalence was detected in The Americas (9.4%), followed by Asia (7.5%), and Europe (3.5%). Overall, the HPV 16 prevalence was calculated to be 6.1%. The highest HPV 18 prevalence was found in Asia (4.8%) followed by the Americas (2.3%) and finally Europe (0.7%). Overall, the HPV 18 prevalence was 3.1%. On all three continents, the calculated prevalence of HPV 16 was higher than for HPV 18 (*P* < .01). Figure 4 depicts the calculated overall HPV prevalence as well as divided by regions and HPV‐genotypes. Tables 2, 3, 4 show the selected studies from Europe, Asia, and America.

**TABLE 2:** Included studies from Europe

Reference	Country	No. of cases	Year	HPV prevalence [%]	Specimen type used	Histological subtypes	HPV types detected
Anantharaman et al^25^	Multiple countries	290	2014	9.7	FFPE, fresh frozen	SCC/AC/others	11, 16, 51, and 58
Argyri et al^26^	Greece	67	2017	3.0		SCC/AC/others	16 and 53
Carpagnano et al^10^	Italy	89	2011	16.4	FFPE	SCC/AC/others	16, 30, 31, and 39
Ciotti et al^27^	Italy	38	2006	8.0	FFPE, fresh	SCC/AC/others	16 and 18
Coissard et al^28^	France	218	2005	1.8	Fresh frozen	SCC/AC/others	16
Eberlein‐Gonska et al^13^	Germany	55	1992	5.5	Fresh	SCC/AC/others	16
Galvan et al^15^	Italy, United Kingdom	100	2012	0	Fresh frozen	SCC/AC/others	None
Gatta et al^16^	Italy	50	2012	4.0	FFPE	SCC	
Guliani et al^29^	Italy	78	2007	12.8	Fresh frozen	SCC/AC/others	16, 18, 31, and 53
Hennig et al^30^	Norway	22	1999	13.6	FFPE	SCC/AC/others	6
Miasko et al^31^	Poland	94	2004	12.7		SCC/AC/others	
Miasko et al^32^	Poland	40	2001	10.0	FFPE	SCC/AC/others	
Jaworek et al^33^	Czech Republic	80	2020	0	FFPE	SCC/AC/others	None
Papadopoulou et al^34^	Greece	52	1998	40.0	Fresh frozen, FFPE	SCC	6, 11, 16, and 18
Podsiadlo et al^35^	Poland	33	2012	3.0	Fresh	NSCLC/SCLC	120
Ramqvist, et al^36^	Sweden	87	2019	0	FFPE	AC/others	None
Sagerup et al^37^	Norway	334	2014	3.9	Fresh frozen	SCC/AC/others	11, 16, 33, and 66
Sarchianaki et al^38^	Greece	100	2014	19.0	FFPE	SCC/AC/others	6, 11, 16, 18, 31, 33, and 59
Shamanin et al^39^	Germany	85	1994	0	Fresh frozen	SCC/AC/others	None
Spandidos et al^40^	Greece	99	1996	15.0	FFPE	SCC/AC/others	11, 16, 18, and 33
Syrjanen et al^41^	Finland	77	2012	5.2	FFPE, archival tissue	SCC/AC/others	6 and 16
Van Boerdonk et al^42^	Netherlands	211	2013	0	FFPE, archival tissue	SCC/AC/others	None
Thomas et al^43^	France	31	1995	16.0	Fresh frozen	SCC/AC/others	6, 11
Welt et al^44^	Germany	38	1997	0	FFPE	SCC/SCLC	None
Zafer et al^45^	Turkey	40	2004	5.0	Fresh frozen	SCC/AC/others	18
Total		2393					

**TABLE 3:** Included studies from Asia

Reference	Country	No. of cases	Year	HPV prevalence [%]	Specimen type used	Histologic subtypes	HPV types detected
Aguayo et al^46^	Pakistan, China	60	2010	13.0	FFPE	SCC/AC/others	16
Baba et al^47^	Japan	57	2010	19.3	FFPE	SCC/AC	6, 16, 18, and 33
Cheng et al^11^	Taiwan	141	2004	38.3		SCC/AC	6 and 11
Cheng et al^12^	Taiwan	141	2001	54.6	FFPE, fresh frozen	SCC/AC	16 and 18
Fan et al^14^	China	262	2015	8.4	FFPE	SCC/AC	16, 18, 31, and 58
Goto et al^48^	Multiple countries	304	2011	7.9	FFPE	SCC/AC	6, 11, 16, and 18
Halimi et al^49^	Iran	30	2011	10.0	FFPE	SCC	
Hartley et al^50^	Lebanon	20	2015	0	FFPE	SCLC	none
He et al^51^	China	140	2019	9.3	Fresh frozen	SCC/AC/others	16 and 18
Hirayasu et al^52^	Japan	73	1996	60.3	FFPE	SCC	6, 16, and 18
Hiroshima et al^53^	Japan	22	1999	4.5	FFPE	AC	16
Ilahi et al^54^	Pakistan	9	2016	11.1	FFPE	SCC/AC/others	16
Isa et al^55^	Japan	96	2015	1.0	FFPE	SCC/AC/others	6
Ito et al^56^	Japan	901	2014	0.9		SCC/AC/others	
Iwakawa et al^57^	Japan	297	2010	0	Fresh frozen	AC	none
Jafari et al^58^	Iran	50	2013	18.0	FFPE	SCC/AC/others	6 and 18
Jain et al^59^	India	40	2005	5.0	Fresh frozen	SCC/AC/others	18
Kato et al^60^	Japan	42	2012	16.7	FFPE	SCC/AC/others	16 and 58
Kawaguchi et al^61^	Japan	876	2016	0.3	FFPE	SCC/AC	16, 62, and 66
Kinoshita et al^62^	Japan	36	1995	8.0	FFPE, fresh frozen	SCC/AC	18
Lee et al^63^	Korea	233	2016	0	FFPE	SCC/AC	none
Li et al^17^	China	50	1995	32.0	FFPE, fresh frozen	SCC/AC/others	16 and 18
Lin et al^64^	Taiwan	57	2005	50.9	FFPE	SCC/AC	16 and 18
Lu et al^18^	China	72	2016	45.8	FFPE	SCC/AC	16 and 18
Miyagi et al^65^	Japan	121	2001	33.9	FFPE	SCC/AC	6, 16, and 18
Nadji et al^19^	Iran	129	2007	25.6	FFPE	SCC/AC/others	6, 11, 26, 31, 16, and 18
Ogura et al^66^	Japan	29	1993	10.3	Fresh frozen	SCC	16 and 18
Park et al^67^	Korea	112	2007	53.6		AC/NSCLC	16, 18, and 33
Wang et al^68^	Taiwan	153	2006	45.1	Fresh	SCC/AC	16 and 18
Wang et al^21^	China	313	2008	44.1	Fresh frozen	SCC/AC	16 and 18
Wang et al^22^	China	45	2010	42.2	Fresh frozen	SCC	16 and 18
Xing et al^69^	China	49	1993	14.2	FFPE	SCC	6, 11, and 16
Yang et al^70^	China	50	1998	26.0	FFPE	SCC	16
Yu et al^23^	China	180	2015	55.6	FFPE	SCC/AC/SCLC	16 and 18
Zhang et al^24^	China	68	2009	44.1	Fresh frozen	SCC, AC	16 and 18
Zhang et al^71^	China	104	2010	17.3	FFPE	SCC/AC/others	16
Total		5362					

**TABLE 4:** Included studies from The Americas

Reference	Country	No. of cases	Year	HPV prevalence [%]	Specimen type used	Histologícal subtypes	HPV types detected
Aguayo et al^72^	Chile	69	2007	29.0	FFPE	SCC/AC/others	6, 16, 18, 31, and 45
Badillo‐Almaraz et al^73^	Mexico	39	2013	41.0		SCC/AC	16 and 18
Bohlmeyer et al^74^	USA	34	1998	5.9	FFPE	SCC	18
Cardona et al^75^	Multiple South American countries	132	2013	39.4	FFPE	AC	16
Carlson et al^76^	USA	12	2007	0	FFPE	SCLC	None
Castillo et al^77^	Peru/Colombia/Mexico	36	2006	28.0	FFPE	SCC/AC/others	16, 18, and 33
de Oliveira et al^78^	Brazil	63	2018	52,4	FFPE	SCC/AC/others	16 and 18
Garcia Falcone et al^79^	Argentina	40	2017	25.0	FFPE	SCC	16 and 18
Joh et al^80^	USA	30	2010	16.7	FFPE	SCC/AC/others	11, 16, and other
Koshiol et al^81^	USA	399	2011	0	FFPE, ethanol fixed	SCC/AC	none
Mehra et al^82^	USA	36	2013	11.0		SCC/AC	16 and 18
Pillai et al^83^	USA	208	2013	14.9	FFPE	NSCLC	16 and 18
Rezazadeh et al^84^	USA	16	2008	25.0	FFPE	NSCLC	11 and 16
Robinson et al^20^	USA	70	2016	42.9	Fresh frozen	SCC/AC	16, 18, 39, 44, 51, 52, and 68
Silva et al^85^	Brazil	62	2019	0	FFPE	SCC/AC/others	None
Suh et al^86^	USA	48	2010	2.0	FFPE	SCC	No data
Yanagawa et al^87^	Canada	336	2013	1.5	FFPE	SCC/AC	16
Total		1630					

### 3.5 Histology and HPV prevalence

Only the information on primary squamous cell carcinoma (SCC) and primary adeno carcinoma (AC) of the lung was collected. In the remaining cases, it was neither one of them or the histological subtype was not detailed. There were 2750 cases of SCC and 2887 cases of AC. In total, 29.3% of the included cases were squamous cell carcinomas and 30.8% were adenocarcinomas.

The overall HPV prevalence in SCC (n = 492) was calculated to be 17.9%. The highest prevalence was calculated in Asia (28.8%), followed by The Americas (10.0%), and Europe (5.1%).

The overall HPV prevalence in adenocarcinomas (n = 265) was calculated to be 9.2%. In contrast, the highest HPV prevalence in AC was calculated in the Americas (11.1%), followed by Asia (10.4%), and Europe (6.0%).

**FIGURE 5:**
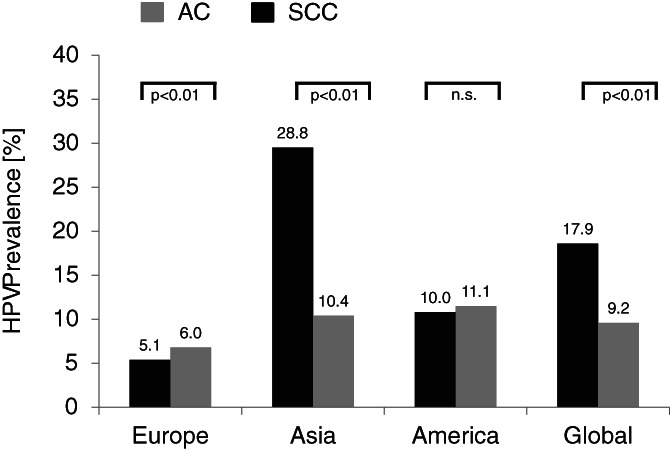
HPV prevalence in SCC vs AC. There was no statistically significant difference between the HPV prevalence in SCC and AC in the studies from America (*P* = .78). Statistically significant differences were found in studies from Asia (*P* < .01) and Europe (*P* < .01). On a global observation HPV prevalence in SCC was significantly higher (*P* < .01) when compared to AC

When the HPV prevalences of SCC and AC are compared, the difference is statistically highly significant (*P* < .01), which is due to a significantly higher HPV prevalence in SCC (*P* < .01) in Asia, whereas no differences in prevalence were found in The Americas and Europe based on histological subtypes of lung cancer. Figure 5 shows the calculated HPV prevalences.

## 4 DISCUSSION

Growing evidence supports the association between HPV‐infection and lung cancer but the relationship is still debatable. The aim of the present study was to conduct a systematic database and literature review by means of a molecular biology based clear definition of HPV positivity and lung cancer. Selection was restricted to studies with lung tissue analysis and PCR‐based confirmation of HPV‐positivity to take advantage of the high specificity and sensitivity of the diagnostic approach. Data of over 9000 lung cancer patients were analyzed, which underlines the robustness of the dataset generated.

The included case‐control studies demonstrated an absolute risk increase of 22% (95% CI: 12%‐33%) in lung cancer patients of being HPV positive, which resulted in a 4.7‐fold (95% CI: 2.7%‐8.4%) increase in the likelihood to detect HPV in patients diagnosed with lung cancer compared to healthy controls regardless of histology or stage of tumor disease.

The meta‐analysis shows that the average HPV infection rate of lung cancer in the world is 13.5% based on PCR‐based assays only. PCR was permitted as the sole method to minimize differences in prevalence related to significant disparities in methodological sensitivity and specificity. Significant regional differences in HPV prevalence in lung cancer patients were found being highest in Asia with 16.6% and lowest in Europe with 7.0%. In addition, the data demonstrate a higher overall HPV prevalence in lung cancer with squamous cell histology, which is mainly due to a significantly higher HPV prevalence in squamous cell carcinoma in Asian regions since this difference was not found in squamous cell carcinoma and adenocarcinoma diagnosed in Europe and America. Most likely, the intriguing different geographic patterns of HPV prevalence in lung cancer are related to the regional differences of the HPV infection itself.

Furthermore, if HPV infection was found, high‐risk genotypes with oncogenic potential were prevalently identified as well. With focus on the most common high‐risk genotypes, overall HPV genotype 16 was the most frequent genotype reported with a twofold higher prevalence compared to HPV genotype 18. With some minor modification, similar findings were reported in all different continents analyzed. These findings additionally support the hypothesis that HPV infections with high‐risk oncogenic potential significantly increase the risk of lung cancer and provide new possibilities in the future in the prevention of lung cancer by means of prophylactic vaccines for the carcinogenic HPV‐16/18 infections.^88^

The pathogenesis of HPV infection in thoracic visceral lungs is still incompletely understood. Blood based transmission through cervical lesion to the lung, high‐risk sexual behavior, and airborne transmission to the lungs have been discussed.^89^ HPV oncogenes (eg, HPV E6 and HPV E7) are known to regulate the expression of multiple target genes and proteins such as p53, pRb, HIF‐1α, VEGF, IL‐6, IL‐10, Mcl‐1, Bcl‐2, cIAP‐2, EGFR, FHIT, hTERT, HER‐ 2, ROS1, and AhR, which can facilitate lung cell proliferation, angiogenesis, and cell immortalization by means of various signaling pathways.^89^

The data of the present study provide evidence for a possible relationship between lung cancer and HPV infection, but the study fails to show a high causal interference since no longitudinal data derived from cohort studies or nested case‐control studies are given. In addition, cofounders of possible importance such as smoking status, gender, age, immunosuppressive co‐medications, oncogenic driver mutations, and estrogenic signaling pathways have not been taken into considerations, which limit the value of the results reported. Furthermore, not all HPV subtypes were assessed due to missing specification in many studies, and no transcriptional activity of the HPV genotypes found was included in the meta‐analysis. Since only PCR was included as HPV detection method but this not being the only way to detect HPV, which can potentially bias the study's results further.

In conclusion, our systematic review provides evidence that HPV infection might increase the risk of developing lung cancer. Whereby relevant regional differences with respect to prevalence and histological subtypes were found with a predominance of squamous cell carcinoma. Consistently, our results support the assumption that the high‐risk genotypes HPV 16 and 18 are risk factors for lung cancer. If the understanding of the process of HPV‐related carcinogenesis in lung cancer could be further elucidated by larger prospective studies, this would facilitate the development of efficient HPV‐targeted prevention strategies.

## CONFLICT OF INTEREST

The authors have stated explicitly that there are no conflicts of interest in connection with this article.

## AUTHOR CONTRIBUTIONS

J.K., H.K., M.K., and C.S. provided substantial contributions to the conceptualization of the study. J.K., H.K., W.D., F.Z., and C.S. designed the methodology and were involved in data curation. J.K., W.D., V.F., M.K., F.K., and C.S. wrote the inital draft of the manuscript. All authors critically reviewed the manuscript, and approved the final version for publication.

## ETHICAL STATEMENT

Not applicable.

## Supporting information

Appendix 1: Search strategies.

## Data Availability

The data that support the findings of this study are available in the digital databases Embase (via Ovid, 1974–present), MEDLINE (via Ovid, 1946–present), Cochrane Library (Cochrane Database of Systematic Reviews, Database of Abstracts of Reviews of Effect, Cochrane Central Register of Controlled Trials, Health Technology Assessment Database, NHS Economic Evaluation Database; from inception to present) and Science Citation Index Expanded (Web of Science, 1965–present) as well as the search engine Google Scholar (using Anne‐Wil Harzing's “Publish or Perish” program available from https://harzing.com/resources/publish‐or‐perish).
