# Supplementary material for: HPV and lung cancer: A systematic review and meta‐analysis
Source: Cancer Rep (Hoboken). 2021 Feb 23;4(4):e1350. doi: 10.1002/cnr2.1350 (PMC8388180; doi:10.1002/cnr2.1350)
Supplement: Supplementary file 1 — Appendix 1: Search strategies. [file CNR2-4-e1350-s001.docx]

Appendix 1: Search strategies

HPV and Lung Cancer: A systematic review and meta-analysis

======================================================================

Authors: Julia Karnosky; Wolfgang Dietmaier; Helge Knuettel; Viola Freigang; Myriam Koch; Franziska Koll; Florian Zeman, Christian Schulz

Searches as last run on 06 February 2020.

## Embase (Ovid)

Database: Embase <1974 to 2020 February 05>

Search Strategy:

--------------------------------------------------------------------------------

1 exp lung cancer/

2 ((lung* or pulm* or bronch* or alveol*) adj3 (cancer* or carcinoma* or adenocarcinoma* or neoplas* or malignan* or tumor* or tumour* or blastoma* or dysplas* or squamous or scc* or oat cell or small cell or non-small cell or nonsmall cell)).ti,ab,kw.

3 multiple pulmonary nodule*.ti,ab,kw.

4 pancoast*.ti,ab,kw.

5 (nsclc or sclc).ti,ab,kw.

6 or/1-5

7 exp papillomaviridae/

8 exp papillomavirus infection/

9 human papillomavirus dna test/

10 (papillomavir* or papilloma vir* or alphapapillomavir* or alphapapilloma vir* or hpv).ti,ab,kw.

11 or/7-10

12 6 and 11

## MEDLINE (Ovid)

Database: Ovid MEDLINE(R) ALL <1946 to February 05, 2020>

Search Strategy:

--------------------------------------------------------------------------------

1 exp Lung Neoplasms/

2 ((lung* or pulm* or bronch* or alveol*) adj3 (cancer* or carcinoma* or adenocarcinoma* or neoplas* or malignan* or tumor* or tumour* or blastoma* or dysplas* or squamous or scc* or oat cell or small cell or non-small cell or nonsmall cell)).ti,ab,kf.

3 multiple pulmonary nodule*.ti,ab,kf.

4 pancoast*.ti,ab,kf.

5 (nsclc or sclc).ti,ab,kf.

6 or/1-5

7 exp papillomaviridae/

8 Papillomavirus Infections/

9 Human Papillomavirus DNA Tests/

10 (papillomavir* or papilloma vir* or alphapapillomavir* or alphapapilloma vir* or hpv).ti,ab,kf.

11 or/7-10

12 6 and 11

## Cochrane Library

ID Search

#1 ((lung* or pulm* or bronch* or alveol*) near/3 (cancer* or carcinoma* or adenocarcinoma* or neoplas* or malignan* or tumor* or tumour* or blastoma* or dysplas* or squamous or scc* or oat next cell or small next cell or non-small next cell or nonsmall next cell)):ti,ab,kw

#2 (multiple next pulmonary next nodule* or pancoast* or nsclc or sclc):ti,ab,kw

#3 #1 or #2

#4 (papillomavir* or papilloma next vir* or alphapapillomavir* or alphapapilloma next vir* or hpv):ti,ab,kw

#5 #3 and #4

## Science Citation Index Expanded (SCI-EXPANDED; Web of Science)

TS=((lung* or pulm* or bronch* or alveol*) NEAR/4 (cancer* or carcinoma* or adenocarcinoma* or neoplas* or malignan* or tumor* or tumour* or blastoma* or dysplas* or squamous or scc* or "oat cell" or "small cell" or "non-small cell" or "nonsmall cell"))

TS=("multiple pulmonary nodule*" or pancoast* or nsclc or sclc)

#1 OR #2

TS=(papillomavir* or "papilloma vir*" or alphapapillomavir* or "alphapapilloma vir*" or hpv)

#3 and #4

## Google Scholar

Search terms (field _Keywords_ in program _Publish or Perish_):

lung|pulmonary|bronchial|alveolar cancer|carcinoma|adenocarcinoma|neoplasm|malignant|tumor|tumour|blastoma|dysplasia|squamous|scc|"oat cell"|"small cell"|"non-small cell"|"non small cell"|nsclc|sclc papillomavirus|hpv|papillomaviridae|alphapapillomavirus

## WHO's International Clinical Trials Registry Platform

lung* AND papillomavir* OR lung* AND papilloma-vir* OR lung* AND hpv OR pulm* AND papillomavir* OR pulm* AND papilloma-vir* OR pulm* AND hpv OR bronch* AND papillomavir* OR bronch* AND papilloma-vir* OR bronch* AND hpv OR alveol* AND papillomavir* OR alveol* AND papilloma-vir* OR alveol* AND hpv

## ClinicalTrials.gov

(lung OR pulmonary OR bronchial OR alveolar OR nsclc OR sclc) AND (papillomavirus OR hpv)

## EU Clinical Trials Register

(lung* OR pulm* OR bronch* OR alveol* OR nsclc OR sclc) AND (papillomavir* OR hpv)
